# The Emulsifying Properties, In Vitro Digestion Characteristics and Storage Stability of High-Pressure-Homogenization-Modified Dual-Protein-Based Emulsions

**DOI:** 10.3390/foods12224141

**Published:** 2023-11-15

**Authors:** Meishan Wu, Xiaoye He, Duo Feng, Hu Li, Di Han, Qingye Li, Boya Zhao, Na Li, Tianxin Liu, Jing Wang

**Affiliations:** 1 Institute of Food and Nutrition Development, Ministry of Agriculture and Rural Affairs, Beijing 100081, China; 2 The Key Lab of Food Resources Monitoring and Nutrition Evaluation, Ministry of Agriculture and Rural Affairs, Beijing 100081, China

**Keywords:** dual protein, high pressure homogenization, emulsifying properties, in vitro digestion, storage stability

## Abstract

The droplet size, zeta potential, interface protein adsorption rate, physical stability and microrheological properties of high-pressure-homogenization (HPH)-modified, dual-protein-based whey–soy (whey protein isolate—soy protein isolate) emulsions containing different oil phase concentrations (5%, 10% and 15%; *w*/*w*) were compared in this paper. The in vitro digestion characteristics and storage stability of the dual-protein emulsions before and after HPH treatment were also explored. The results show that with an increase in the oil phase concentration, the droplet size and interface protein adsorption rate of the untreated dual-protein emulsions increased, while the absolute value of the zeta potential decreased. When the oil phase concentration was 10% (*w*/*w*), HPH treatment could significantly reduce the droplet size of the dual-protein emulsion, increase the interface protein adsorption rate, and improve the elasticity of the emulsion. Compared with other oil phase concentrations, the physical stability of the dual-protein emulsion containing a 10% (*w*/*w*) oil phase concentration was the best, so the in vitro digestion characteristics and storage stability of the emulsions were studied. Compared with the control group, the droplet size of the HPH-modified dual-protein emulsion was significantly reduced after gastrointestinal digestion, and the in vitro digestibility and release of free amino groups both significantly increased. The storage stability results show that the HPH-modified dual-protein emulsion showed good stability under different storage methods, and the storage stability of the steam-sterilized dual-protein emulsion stored at room temperature was the best. These results provide a theoretical basis for the development of new nutritional and healthy dual-protein liquid products.

## 1. Introduction

The oil-in-water (O/W) emulsion system is a typical form of foods such as milk, mayonnaise and milk beverages [1]. In an O/W emulsion, the oil exists as a dispersed phase in the form of small droplets suspended in water (continuous phase) [2]. The choice of natural proteins as emulsifiers to prepare O/W emulsions has been attracting the interest of researchers [3,4,5]. As a kind of amphiphilic interfacial active molecule, protein can rapidly absorb at the O/W interface, which changes the molecule’s structure; the hydrophobic and hydrophilic groups combine with the oil phase and water phase separately, reducing the surface tension and forming a viscoelastic interface film [6].

In recent years, in order to solve the shortage of protein resources caused by global population growth and promote environmental sustainability, improving the development and utilization of high-quality plant protein resources and researching the partial replacement of animal protein with plant protein in formulated products have gradually become hot research topics [7,8]. A “dual-protein”, referring to an edible protein source obtained from natural, high-quality plant protein such as soy protein and animal protein such as whey protein, which was discovered by studying the dose–effect relationship and the mechanisms of synergism and interactions of animal–plant mixed proteins, has a complete range of amino acids and is rich in essential amino acids. Its high nutritional value and bioavailability play important roles in maintaining physical health and promoting body growth. Preliminary findings demonstrated that a dual protein had significant nutritional quality improvement in hematopoietic reconstruction, immune function, muscle level, lipid metabolism, and osteoporosis [9,10,11]. Whey protein isolate (WPI) and soy protein isolate (SPI) are the main animal–plant protein ingredients in dual proteins; both have excellent functional processing properties such as emulsification, gelation and foaming, which are widely used in food processing [12,13]. However, commercial SPI always exhibits inferior solubility and emulsifying properties because of its partial denaturation during processing and cannot form a stable O/W emulsion structure [14]. Therefore, it is necessary to study suitable modification methods for improving the emulsifying properties of dual proteins, which has profound significance for improving their application in food processing.

High-pressure homogenization (HPH) is an emerging processing technology that has been widely used in food processing. Originally, HPH was only used to homogenize complex liquid products such as fruit and vegetable juices to enhance their rheological properties and physical stability as a standardization step [15,16]. The working principle can be generally summarized as follows: when a liquid material flows quickly through a homogeneous valve gap with a special internal structure, it is subjected to mechanical forces such as high-speed shear, shock, and the cavitation phenomenon, which crush and break the large liquid particles into small ones [17,18]. HPH is widely used to prepare protein-stabilized emulsions, and suitable homogenization pressure could improve the stability of a protein emulsion by reducing the droplet size and enhancing the rheological and interfacial properties of the emulsion. Ma et al. found that HPH promoted the adsorption of cod protein molecules at the O/W interface, and the elastic modulus (G’) of the emulsion interface film significantly increased [19]. Fernandez-Avila et al. observed that 100 MPa and 200 MPa treatments could allow more SPI to be loaded onto the O/W interface, thereby improving the emulsion’s oxidative stability [20]. Melchior et al. found that the oil-holding capacity of a pea protein emulsion improved as the HPH intensity increased, and the in vitro digestibility performed best with a 70 MPa treatment, comparable to original pea protein [21].

HPH-modified protein emulsions have been extensively studied, while dual-protein emulsion modification via HPH has not been deeply researched so far. In this study, the structure and interfacial properties of HPH-modified dual-protein emulsions were researched, and their in vitro digestion properties and storage stability were assessed.

## 2. Materials and Methods

### 2.1. Materials

SPI (protein content, 90%) was obtained from IFF Inc. (Shanghai, China). WPI (protein content, 95%) was obtained from Agropur Cooperative (Montreal, QC, Canada). Soy oil was purchased from Yihai Karry Co., Ltd. (Shanghai, China). All reagents were of analytical-grade purity.

### 2.2. Preparation of Protein Solution and HPH Treatment

The protein powder (SPI and WPI) were dispersed in de-ionized water at a total protein concentration of 6% (*w/v*) to achieve SPI, WPI, and dual protein (SPI:WPI = 1:1) solutions, and they were stirred at room temperature (25 °C) for 1.5 h. After that, all the solutions were stored at 4 °C for 12 h to ensure full hydration. After the protein solutions were restored to room temperature, they were filtered using a 0.3 mm sieve and homogenized for three cycles at 60 MPa, using a high-pressure homogenizer (Panda PLUS 2000, GEA Group, Düsseldorf, Germany); each cycle time was approximately 13 s. The high-pressure homogenizer was connected to a condenser to keep the temperature of the protein solutions at 18 ± 1 °C after each HPH treatment cycle. Untreated protein solutions were used as a control.

### 2.3. Emulsion Preparation

The protein emulsions containing 5%, 10% and 15% (*w*/*w*) oil phase concentrations were prepared as follows: soy oil was mixed with the protein solutions at 1:19, 1:9 and 3:17 mass ratios, using a high-speed homogenizer (Ultra Turrax T25 Digital, IKA-Werke GmbH & Co. KG, Stuttgart, Baden-Württemberg, Germany) at 5000 rpm for 1 min. Then, all the O/W protein emulsions were processed via three cycles of HPH treatment at 50 MPa using a high-pressure homogenizer (Panda PLUS 2000, GEA Group, Düsseldorf, Germany).

### 2.4. Dual-Protein Emulsion Measurements

#### 2.4.1. Droplet Size and Zeta Potential of Emulsion

All the protein emulsions were diluted to 0.12 mg/mL using de-ionized water. The droplet size and zeta potential were determined using a nanoparticle size analyzer (Zetasizer Nano-ZS90, Malvern Instrument Co., Ltd., Malvern, UK) at room temperature (25 °C), according to a previous report [22]. The zeta potential was indirectly calculated from the electrophoretic mobility using Henry’s equation of electric field [23]:

(1) $$\frac{U}{E}=\frac{2\varepsilon \zeta F\left(\kappa a\right)}{3\eta }$$

where U/E is the electrophoretic mobility (m^2^s^−1^V^−1^), ζ is the zeta potential (V), ε is the solvent’s dielectric permittivity (kgmV^−2^s^−2^), η is the viscosity (kgm^−1^s^−1^), and F(κa) is Henry’s function (dimensionless), which varies between 1 and 3/2 according to the ratio of the particle size to the Debye length, 1/κ. 

#### 2.4.2. Interfacial Protein Absorption Rate of Emulsions

As described by Sun et al. [24], the interfacial protein adsorption rates (AP%) of the emulsions were measured using the Bradford method, and bovine serum albumin (BSA) was used as a standard. All the protein emulsions were centrifuged at 19,890 rpm for 5 h (4 °C). After centrifugation, the emulsion was separated into two layers; then, the cream phase was removed, and the aqueous phase was filtered through a 0.45 μm filter and diluted to 0.06 mg/mL using de-ionized water. The diluted aqueous phase was mixed with Coomassie brilliant blue G-250 at a volume ratio of 1:5 for 2 min, and the absorbance was measured using an ultraviolet–visible spectrophotometer (UV1780, Shimadzu Corporation, Kyoto, Japan) at 595 nm. By substituting the absorbance into the BSA standard curve, the protein concentration of the aqueous phase was calculated. Finally, the AP% was calculated according to the following equation:

(2) $$AP\%=\frac{C_{0}-C_{1}}{C_{0}}\times 100\%$$

In Equation (2), *C*_0_ and *C*_1_ are the initial protein concentration of the emulsion and the protein concentration of the aqueous phase, respectively.

#### 2.4.3. Physical Stability of Emulsions

A Tubiscan stability analyzer (Tubiscan Tower, Formulaction, Toulouse, France) was used to measure the physical stability of the protein emulsions. According to the previous method reported by Shi et al. [25], a back-scattered light was set to measure at 15 min intervals for 5.75 h, and all the tests were performed at room temperature (25 °C). The changes in the backscattering profiles and Turbiscan stability index (TSI) were calculated using Towersoft 2.0.0.9.

#### 2.4.4. Micro-Rheological Properties of Emulsions

Micro-rheology of the protein emulsions was performed using an optical microrheometer (Rheolaser Master, Formulaction, Toulouse, France). According to the previous method described by Zhu et al. [26], 20 mL of a protein emulsion was added into a glass tube and then placed in the Rheolaser chamber. The emulsion was measured every 5 s for a total of 20 min at room temperature (25 °C). The elasticity index (EI), macro-viscosity index (MVI) and solid-liquid balance (SLB) of the protein emulsion were obtained using a Rheosoft Master 1.4.0.0.

### 2.5. In Vitro Digestion Behavior Analysis of Dual-Protein Emulsions

#### 2.5.1. In Vitro Digestion Treatment

According to the previous method described by Ding et al. [27], a simulation of gastric–intestinal digestion was designed to conduct in vitro static digestion.

Gastric phase: 2 g of NaCl and 7 mL of HCl (37%) were dissolved in 1 L of de-ionized water, and then 3.2 g of pepsin was added to the solution. The prepared simulated gastric fluid (SGF) was mixed with the emulsion at a volume ratio of 1:1, and the pH of the mixture was adjusted to 2.0 using 1.0 M of HCl and 1.0 M of NaOH. Then, the mixture was incubated at 37 °C for 2 h with continuous agitation in a thermostatic water bath (DF-101S, Lichen Experimental Equipment Manufacturing Co., Ltd., Shanghai, China).

Intestine phase: The pH of each sample after gastric digestion was adjusted to 7.0; then, 1.5 mL of salt solution (10 mM of CaCl_2_ and 150 mM of NaCl), 3.5 mL of porcine bile extract solution (54 mg/mL in a phosphate buffer, pH 7.0) and 2.5 mL of trypsin solution (24 mg/mL in a phosphate buffer, pH 7.0) were added to the sample in order. After that, the pH of the mixture was adjusted to 7.0 using 1.0 M of HCl and 1.0 M of NaOH. Then, the mixture was incubated at 37 °C for 2 h with continuous agitation in a thermostatic water bath (DF-101S, Lichen Experimental Equipment Manufacturing Co., Ltd., Shanghai, China).

#### 2.5.2. The Droplet Size and Zeta Potential of the Emulsions during In Vitro Digestion

Digestive samples were taken at 30, 60, 90, and 120 min during gastric digestion and intestinal digestion, respectively, which were treated based on enzyme inactivation. All the samples were diluted to 0.12 mg/mL using de-ionized water, and the droplet size and zeta potential were measured using a nanoparticle size analyzer (Zetasizer Nano-ZS90, Malvern Instrument Co., Ltd., Malvern, UK) at room temperature (25 °C). 

#### 2.5.3. The Free Amino Groups of the Emulsions during In Vitro Digestion 

According to Church et al. [28], the free amino groups (-NH_2_) of the emulsions during in vitro digestion were measured via the ortho-phthalaldehyde (OPA) method, and DL-serine was used as a standard. The OPA solution was made by combining the following reagents and diluted to a final volume of 1 L with de-ionized water: 38.1 g of sodium tetraborate, 1 g of sodium dodecyl sulfate (SDS), 0.8 g of OPA (dissolved in 20 mL of absolute ethanol), and 0.88 g of dithiothreitol (DTT). This reagent was prepared freshly every day. The enzyme inactive samples were taken at 30, 60, 90, and 120 min during gastric digestion and intestinal digestion, respectively, and then they were centrifuged at 19,890 rpm for 3 h (4 °C). After centrifugation, the cream phase was removed, and the aqueous phase was diluted to 0.6 mg/mL using de-ionized water. Then, 400 μL of the diluted aqueous phase was mixed with 3 mL of the OPA solution for 2 min, and the absorbance was measured using an ultraviolet–visible spectrophotometer (UV1780, Shimadzu Corporation, Kyoto, Japan) at 340 nm. By substituting the absorbance into the DL-serine standard curve, the -NH_2_ of the aqueous phase was calculated. 

#### 2.5.4. In Vitro Digestibility of Emulsions

The in vitro digestibility of the emulsions was measured using the Bradford method. The enzyme-inactive samples of all the emulsions were taken at the end of the gastric–intestinal digestion, and they were centrifuged at 19,890 rpm for 3 h (4 °C). After centrifugation, the cream phase was removed, and the aqueous phase was diluted to 0.12 mg/mL using de-ionized water. The diluted aqueous phase was mixed with Coomassie brilliant blue G-250 at a volume ratio of 1:5 for 2 min, and the absorbance was measured using an ultraviolet–visible spectrophotometer (UV1780, Shimadzu Corporation, Kyoto, Japan) at 595 nm. By substituting the absorbance into the BSA standard curve, the protein concentration of the aqueous phase was calculated. Finally, the in vitro digestibility was calculated according to the following equation:

(3) $$In\;vitro\;digestion\%=\frac{C_{0}-C_{1}}{C_{0}}\times 100\%$$

In Equation (3), *C*_0_ and *C*_1_ are the initial protein concentration of the emulsion and the protein concentration of the aqueous phase after in vitro digestion, respectively.

### 2.6. Storage Stability of Dual-Protein Emulsions

All the emulsions were treated with 65 °C pasteurization for 30 min, 95 °C boiled sterilization for 10 min, and 121 °C steam sterilization for 20 min, respectively. Untreated emulsions were used as controls. The control and pasteurization samples were stored at 4 °C, and the boiled-sterilization and steam-sterilization samples were stored at room temperature (25 °C). The storage stability of the emulsions was measured by the changes in the appearances of the emulsions at 0, 7, 14, 21, and 28 d.

### 2.7. Statistical Analysis

All the statistical differences in the data were analyzed using SPSS 26 (IBM Corporation, Armonk, NY, USA) through a one-way analysis of variance (ANOVA), and Origin 2021 was used for data analysis and visualization. The differences were considered significant at *p* < 0.05. 

## 3. Results and Discussion

### 3.1. Droplet Size and Zeta Potential

**Figure 1:**
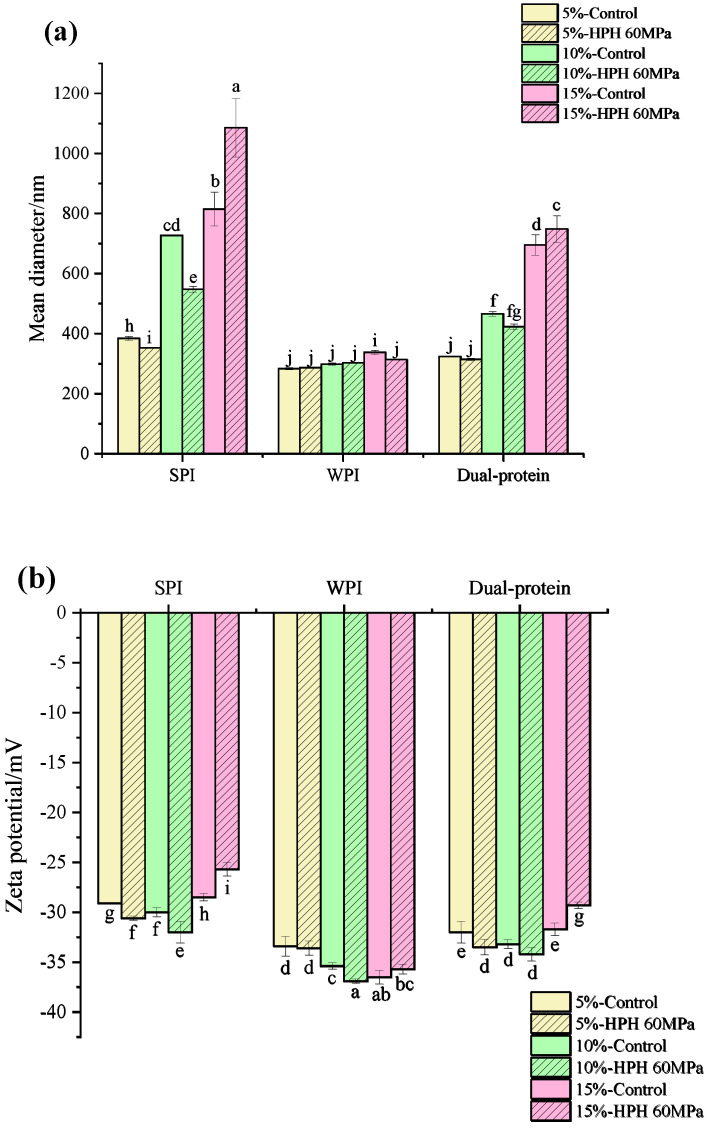

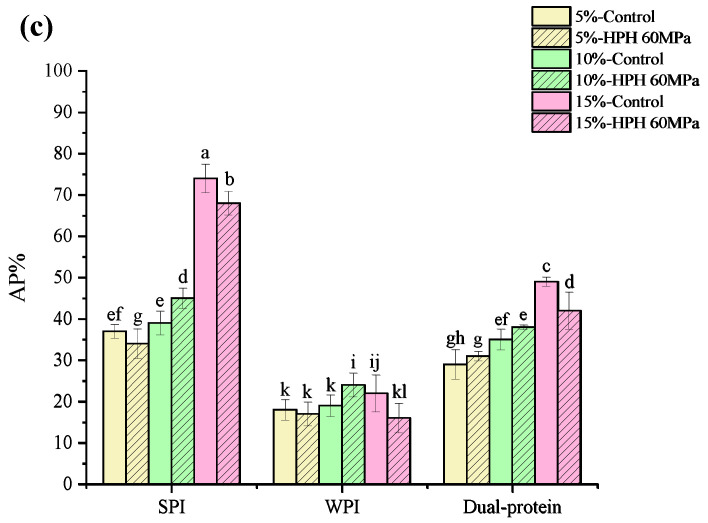
The droplet size (**a**), zeta potential (**b**), and interfacial protein absorption rate (**c**) of protein emulsions containing different oil phase concentrations. Different lowercase letters in Figures (**a**–**c**) indicate significant differences in the different protein emulsions (*p* < 0.05).

The droplet size and zeta potential of a protein emulsion depend on the protein trapped on the surface of the oil, which are important factors affecting the physicochemical properties of emulsion [29]. Figure 1a,b show the droplet size and zeta potential results of the protein emulsions, respectively. It can be seen that as the oil phase concentration rose, the droplet size of the untreated protein emulsion increased significantly, which was because the emulsified oil droplet volume needed to be increased to satisfy the limit of the protein emulsifier absorbed on the surface of the oil. The mean droplet size of the HPH-modified SPI emulsion containing the 10% (*w*/*w*) oil phase concentration decreased obviously from 685 nm to 547 nm, and the absolute value of its zeta potential also increased to the maximum (32 mV). Perhaps because the high shear rate could cut the emulsion droplets into smaller ones during HPH treatment and more negative charges were exposed on the surface of the protein, which increased the electrostatic repulsion between droplets, the stability of the emulsion was enhanced. A similar result demonstrated that HPH played a key role not only in the reduction of the droplet size but also in the particle dispersion, as Song et al. [30] reported before. The same changes took place in the HPH-modified dual-protein emulsion containing a 10% (*w*/*w*) oil phase concentration: the droplet size decreased from 485 nm to 425 nm, and the absolute value of the zeta potential increased to 34.2 mV. The mean droplet size of the HPH-modified WPI emulsion containing a 10% (*w*/*w*) oil phase concentration had no significant difference compared to the control, whereas the absolute value of the zeta potential increased significantly from 35.4 mV to 36.9 mV, indicating that HPH was beneficial in improving the dispersion stability of the WPI emulsion. As the oil phase concentration rose to 15% (*w*/*w*), the mean droplet size of the HPH-modified SPI and dual-protein emulsions both obviously increased to 1085.4 nm and 748.1 nm separately, and the absolute values of the zeta potential decreased, suggesting that excessive oil phase aggregation might have caused the coalescence and flocculation of the emulsion.

### 3.2. Interfacial Protein Absorption Rate 

The AP% at the O/W interface is also one of the most important indexes for measuring the stability of an emulsion [31]. Figure 1c shows the AP% of the protein emulsions. As the oil phase concentration rose, the AP% at the interface of the untreated protein emulsion increased gradually. The AP% of the dual-protein emulsion was lower than the SPI emulsion, but it was much higher than the WPI emulsion, indicating that the existence of SPI could improve the absorption of WPI molecules at the O/W interface. All the AP% values of the HPH-modified protein emulsions improved at the 10% (*w*/*w*) oil phase concentration, which might be attributed to the improvement of the surface hydrophobicity of protein after HPH treatment, promoting the interface’s absorption capacity at this oil phase concentration [32]. The AP% values of the HPH-modified SPI and WPI emulsions only obviously increased compared with the control ones at the 10% (*w*/*w*) oil phase concentration, while the AP% of the HPH-modified dual-protein emulsion increased at both the 5% and 10% (*w*/*w*) oil phase concentrations, indicating that HPH had more positive effect on enhancing the interfacial absorption property of the dual-protein emulsion. However, as the oil phase concentration rose to 15% (*w*/*w*), compared with the control ones, all the AP% values of HPH modified protein emulsions decreased, which might be because the interface film was loaded its maximum absorption capacity, and the aggregation of unloaded oil droplets inhabited the absorption of protein molecules at the O/W interface.

### 3.3. Physical Stability Properties

**Figure 2:**
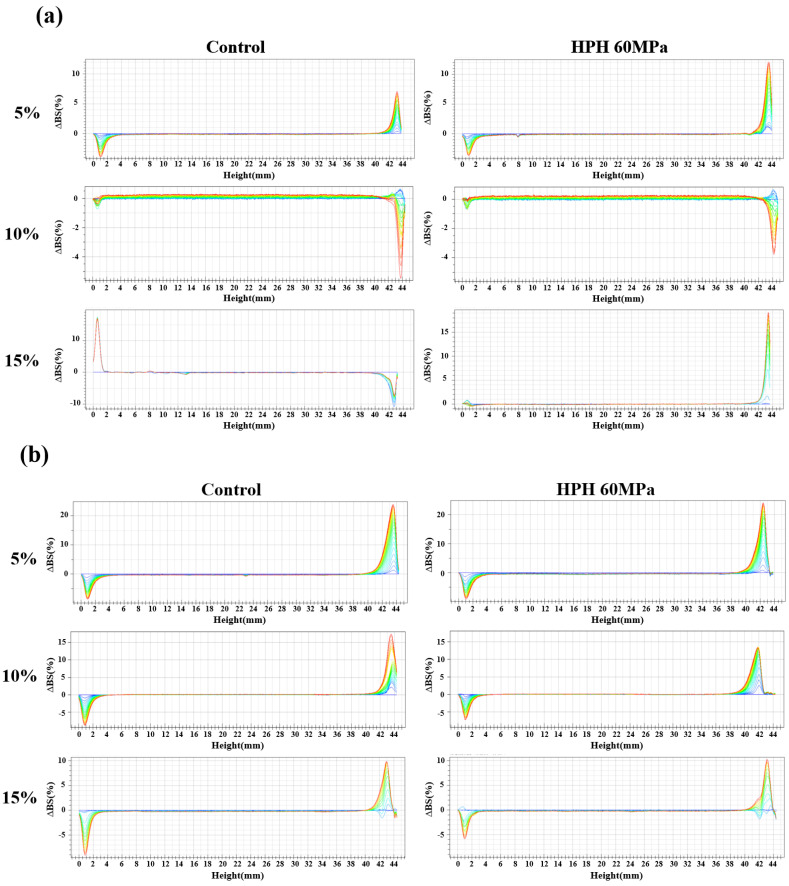

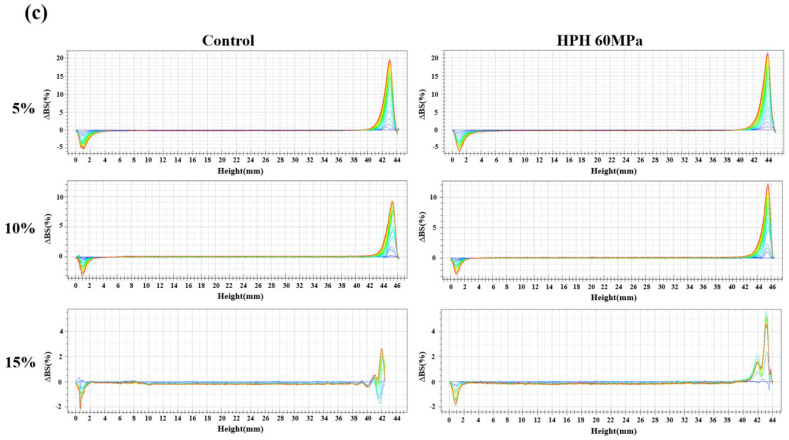
The changes in the backscattering profiles of SPI (**a**), WPI (**b**) and dual-protein (**c**) emulsions containing different oil phase concentrations. The color of the lines in Figures (**a**–**c**) gradually changes from blue to green and eventually to red as time goes on.

The Turbiscan stability analyzer is based on the principle of multiple heavy light scattering, carrying out top-down scanning using transmitted light and backscattered light sources passing through the static sample after a certain period of time to characterize changes in the liquid sample according to the collected spectral line data and analyzing the mechanism of system instability in real time [33]. Figure 2a–c show the backscattering changes of the SPI, WPI, and dual-protein emulsions, respectively. Clearly, the backscattering of the untreated SPI emulsion showed the condition change from floating to sediment as the oil phase concentration rose, whereas the untreated WPI and dual-protein emulsions both showed the floating condition at three oil phase concentrations, and the backscattering of the right side decreased as the oil phase concentration rose, reflecting that the floating phenomenon of the emulsion declined because the increase in the emulsion’s particle size improved the flow resistance of the emulsion, according to what Hebishy et al. [34] reported previously. The backscattering of the untreated dual-protein emulsion was significantly lower than the untreated WPI emulsion at the same oil phase concentration, indicating that the stability of the dual-protein emulsion was better than the WPI emulsion. At the 10% (*w*/*w*) oil phase concentration, the backscattering of the SPI emulsion reduced after HPH treatment, and the same phenomenon could be found in the WPI emulsion, indicating that HPH could improve the physical stability of the SPI and WPI emulsions at this oil phase concentration. However, after HPH treatment, the backscattering of the right side of the dual-protein emulsions slightly increased at three oil phase concentrations; as reported previously, this might be caused by the supramolecular aggregates formed, which may reduce the rate of diffusion and rearrangement on oil–water interface, thus preventing the interface film from forming in time and resulting in the aggravation of floating [35].

The Turbiscan Stability Index (TSI) can reflect the kinetic instability of an emulsion system within a certain time. The smaller the TSI value, the better the system’s physical stability. It was found that the TSI of all the protein emulsions increased gradually with time, and the TSI values of the protein emulsions at the last scanning time are shown in Table 1. The TSI of the untreated SPI emulsion reached a maximum at the 15% (*w*/*w*) oil phase concentration, and the TSI of the untreated dual-protein emulsion was lower than the untreated WPI emulsion at the same oil phase concentration. The HPH-modified SPI emulsion had the lowest TSI at the 10% (*w*/*w*) oil phase concentration, 0.1, while the TSI values of the HPH-modified WPI and dual-protein emulsions both increased. It is worth noting that the TSI of the HPH-modified dual-protein emulsion was still lower than the HPH-modified WPI emulsion at the same oil phase concentration, suggesting that the dual-protein emulsion was more stable than the WPI emulsion after HPH treatment.

### 3.4. Micro-Rheology of Emulsions

**Figure 3:**
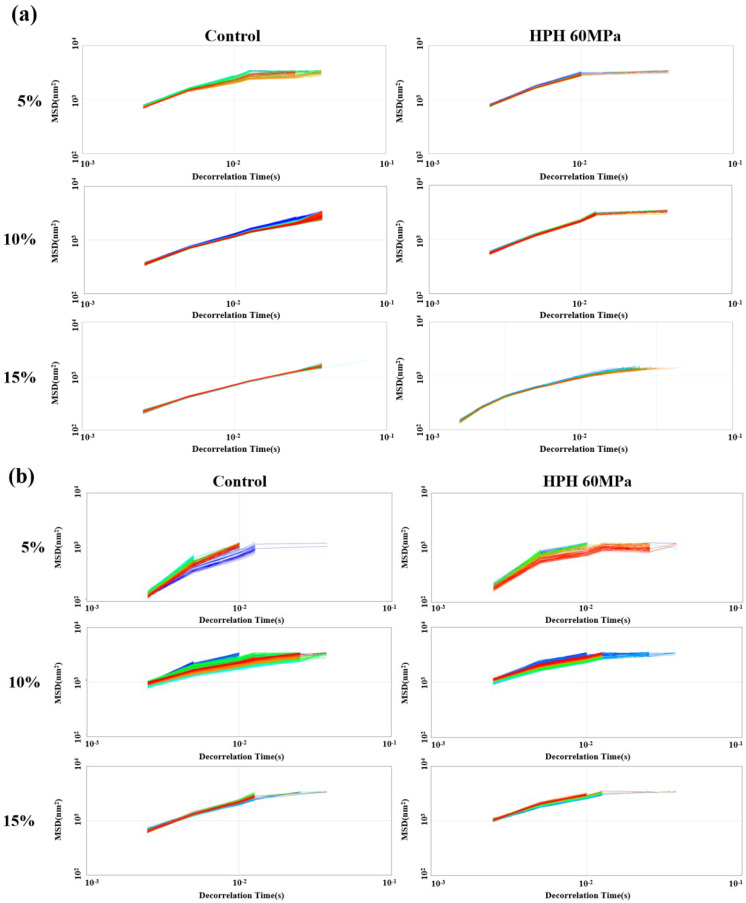

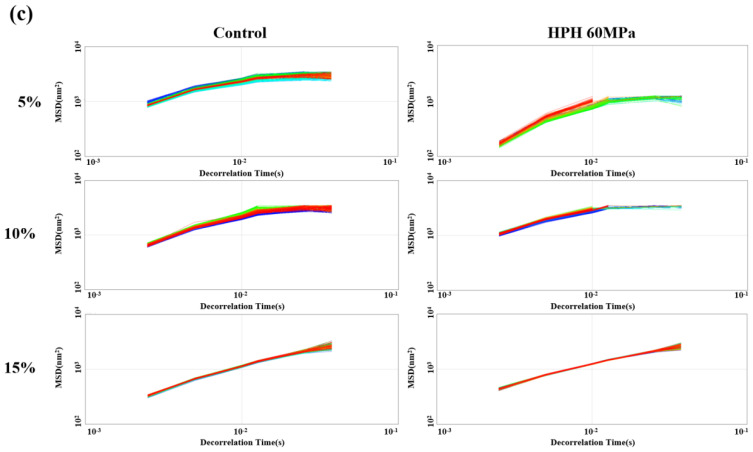
The MSD-time curves of SPI (**a**), WPI (**b**) and dual-protein (**c**) emulsions containing different oil phase concentrations. The color of the lines in Figures (**a**–**c**) gradually changes from blue to green and eventually to red as time goes on.

Micro-rheology can usually be used to trace the motion of colloidal particles at a scale of 0.1–10 μm. By detecting the Brownian motion of droplets, the displacement of particles can be accurately detected, and the micro-rheological behavior of liquids can be analyzed [36]. A Rheolaser Master was used to detect structural and viscoelastic changes in the emulsion system, using the mean square displacement (MSD) of the emulsion droplet as a formula for the de-correlation time. Figure 3a–c show the MSD-time curves of the SPI, WPI and dual-protein emulsions. It can be seen that as the oil phase concentration increased, the MSD curve of the untreated protein emulsion became narrower and denser, indicating that the structure of the emulsion became tighter. The linear relationship between the MSD curve and the de-correlation time of the HPH-modified SPI emulsion was reduced at the 5% and 10% (*w*/*w*) oil phase concentrations, but it returned to a linear relationship again at the 15% (*w*/*w*) oil phase concentration, which mainly reflected that the emulsion’s viscosity became obvious as the oil phase concentration rose. This same phenomenon could be found in the HPH-modified dual-protein emulsion. Regarding the HPH-modified WPI emulsion, it showed a more significant linear relationship at the 10% (*w*/*w*) oil phase concentration, suggesting that the emulsion had strong viscosity at this oil phase concentration. 

**Figure 4:**
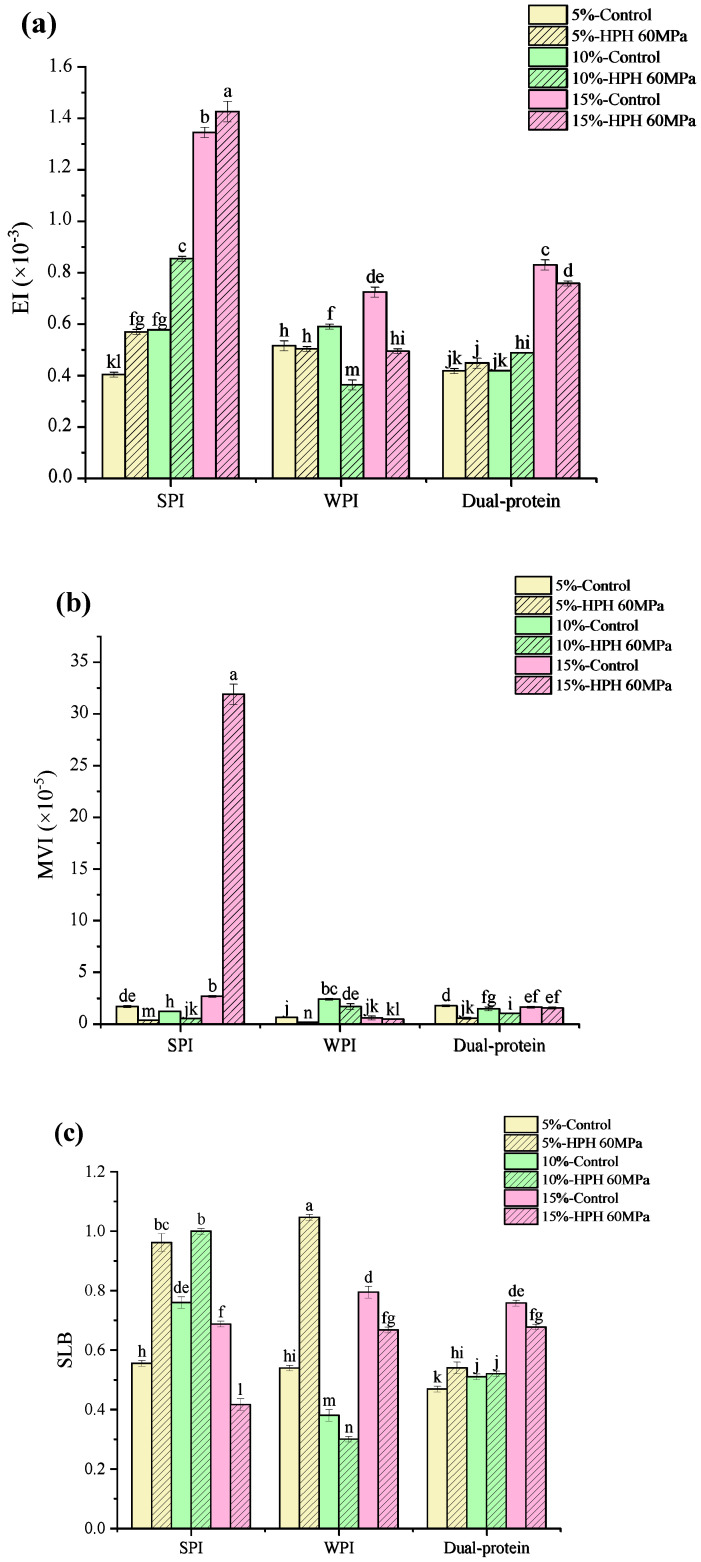
The EI (**a**), MVI (**b**) and SLB (**c**) of protein emulsions containing different oil phase concentrations. Different lowercase letters in Figures (**a**–**c**) indicate significant differences in the different protein emulsions (*p* < 0.05).

The elasticity index (EI) and macroscopic viscosity index (MVI) can reflect the elastic and viscous characteristics of an emulsion, respectively. Figure 4a,b show the EI and MVI of protein emulsions calculated using Rheosoft Master 1.4.0.0 software, respectively. It can be clearly seen that the EI values of the untreated SPI and dual-protein emulsions increased as the oil phase concentration rose, and the EI increased further at the 5% and 10% (*w*/*w*) oil phase concentrations after HPH treatment, indicating that the elasticity of the emulsions was enhanced. On the contrary, the EI of the HPH-modified WPI emulsion declined at three oil phase concentrations, and the elasticity of the emulsion reduced. In addition, the MVI of the protein emulsion decreased after HPH treatment, but the MVI of the HPH-modified SPI emulsion containing the 15% (*w*/*w*) oil phase concentration abnormally increased, indicating that there was abundant oil phase which overflowed, the aggregation of massive oil droplets led to serious damage to the structure of the emulsion, and the viscosity of the emulsion significantly increased after HPH treatment.

The SLB is the slope value of the MSD curve plateau in micro-rheology, which can reflect the solid–liquid equilibrium state of a system. When the SLB < 0.5, the droplet movement speed of the system is slow, which mainly shows elastic solid behavior. When SLB = 0.5, the system is in a solid–liquid equilibrium state. When 0.5 < SLB < 1, viscous liquid behavior is mainly manifested. When SLB > 1, sediment occurs [37]. Figure 4c shows the SLB of the protein emulsions, calculated using Rheosoft Master 1.4.0.0 software. The SLB values of the untreated SPI and WPI emulsions were both between 0.5 and 1, while the SLB of the untreated dual-protein emulsion containing the 5% (*w*/*w*) oil phase concentration was slightly lower than 0.5, showing mild solid behavior. After HPH treatment, the SLB of the SPI emulsion containing the 10% (*w*/*w*) oil phase concentration and the WPI emulsion containing the 5% (*w*/*w*) oil phase concentration both increased to beyond 1, and a slight sediment existed in the emulsions; additionally the SLB of the SPI emulsion containing the 15% (*w*/*w*) oil phase concentration and the WPI emulsion containing 10% (*w*/*w*) decreased obviously to 0.41 and 0.36, respectively, and the structures of the emulsions tended to be more solid. However, the SLB of the HPH-modified dual-protein emulsions were maintained between 0.5 and 1 at the three oil phase concentrations, indicating that the dual-protein emulsion had significant viscous liquid characteristics compared to a single-protein emulsion after an HPH treatment.

In summary, the HPH-modified dual-protein emulsion containing the 10% (*w*/*w*) oil phase concentration had the better diffusion stability, the AP% at the O/W interface increased, and the emulsion’s elasticity was enhanced, showing a more significant solid–liquid equilibrium than a single-protein emulsion. Therefore, the HPH-modified dual-protein emulsion containing the 10% (*w*/*w*) oil phase concentration was selected as the follow-up research object.

### 3.5. In Vitro Digestion Properties of Emulsion

#### 3.5.1. Droplet Size and Zeta Potential during In Vitro Digestion

**Figure 5:**
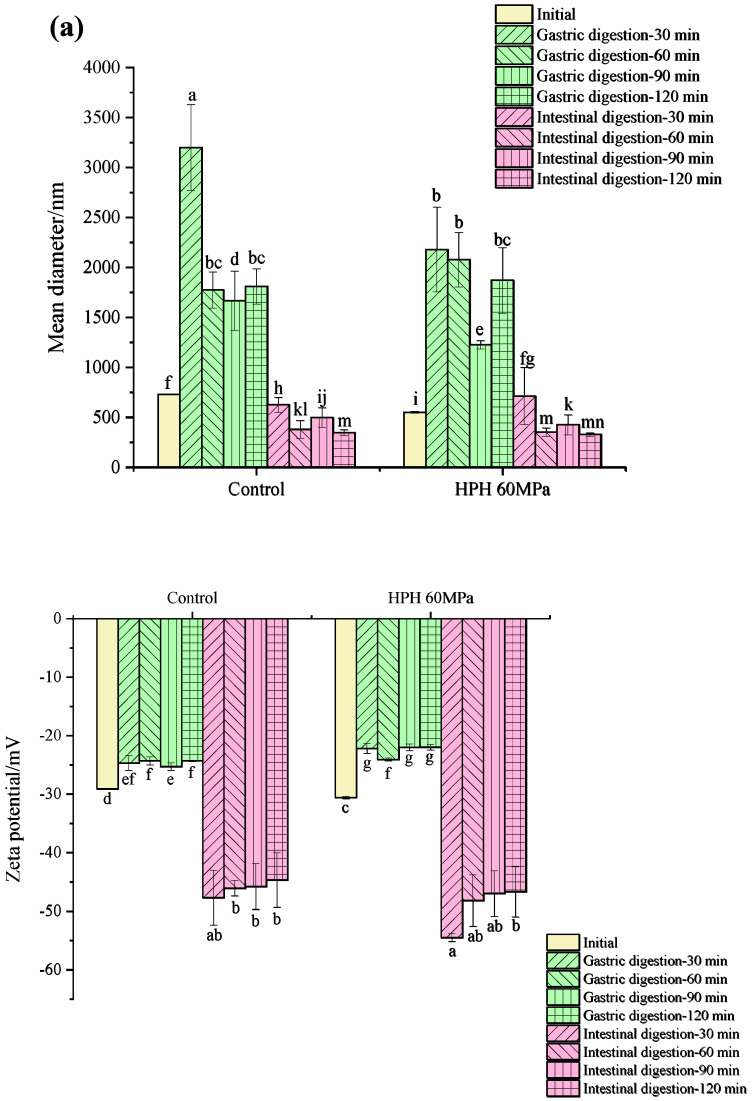

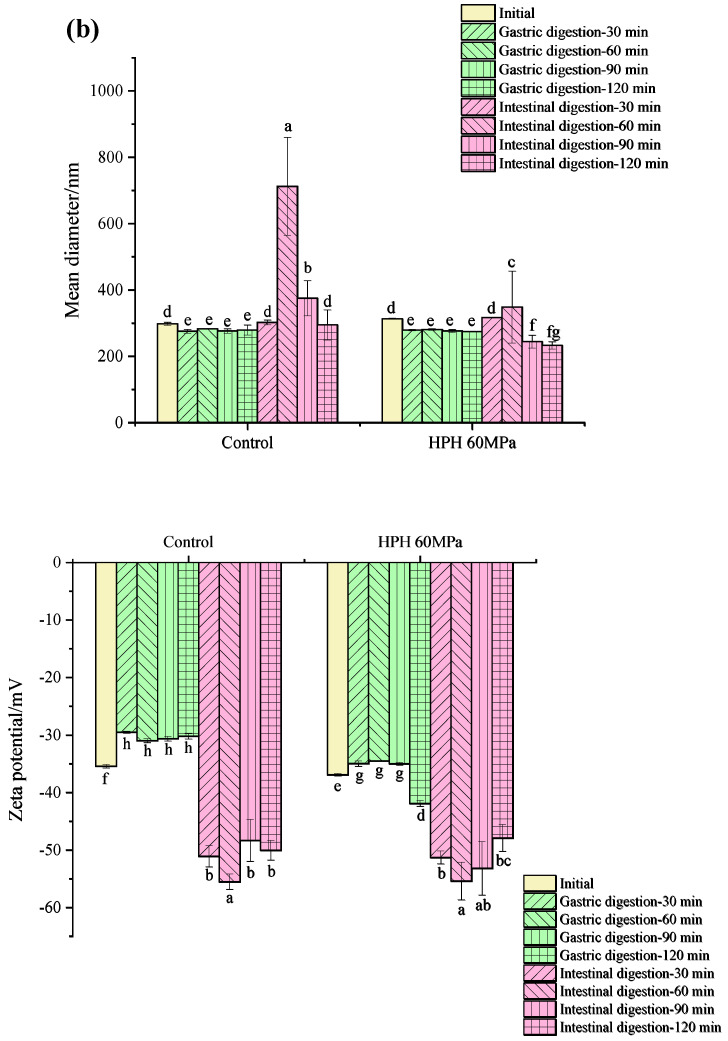

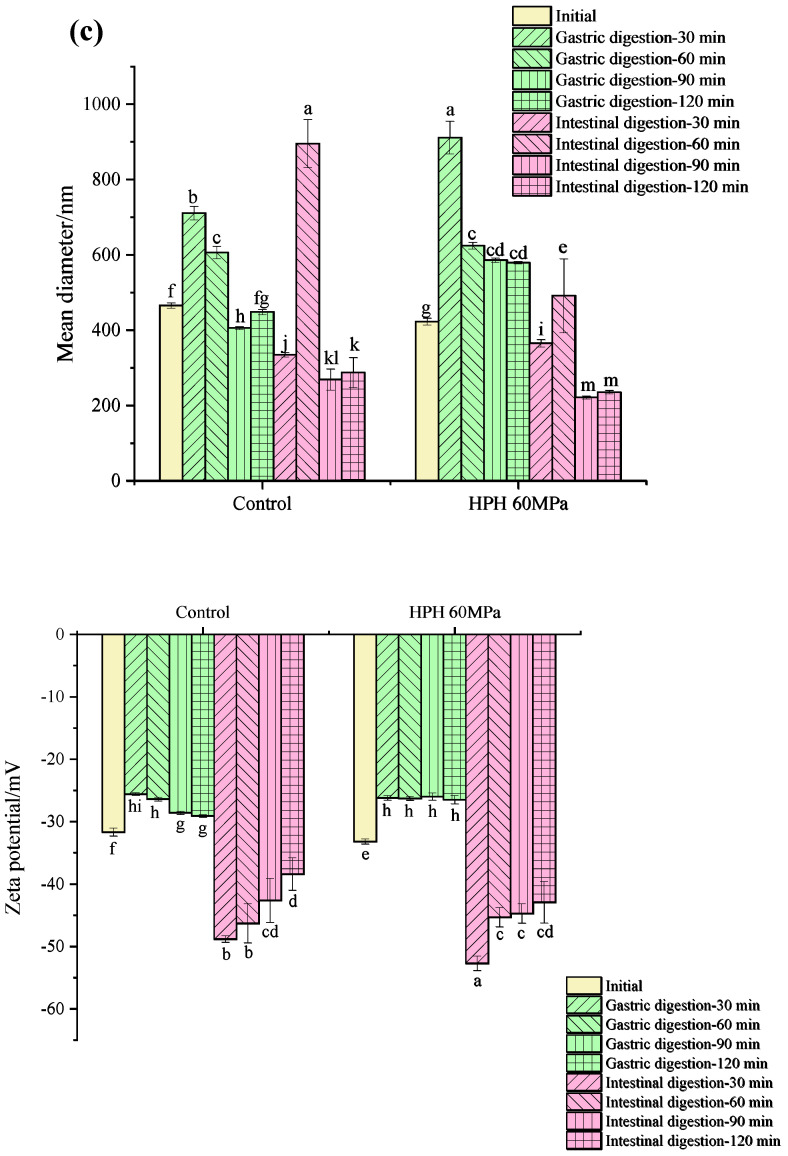

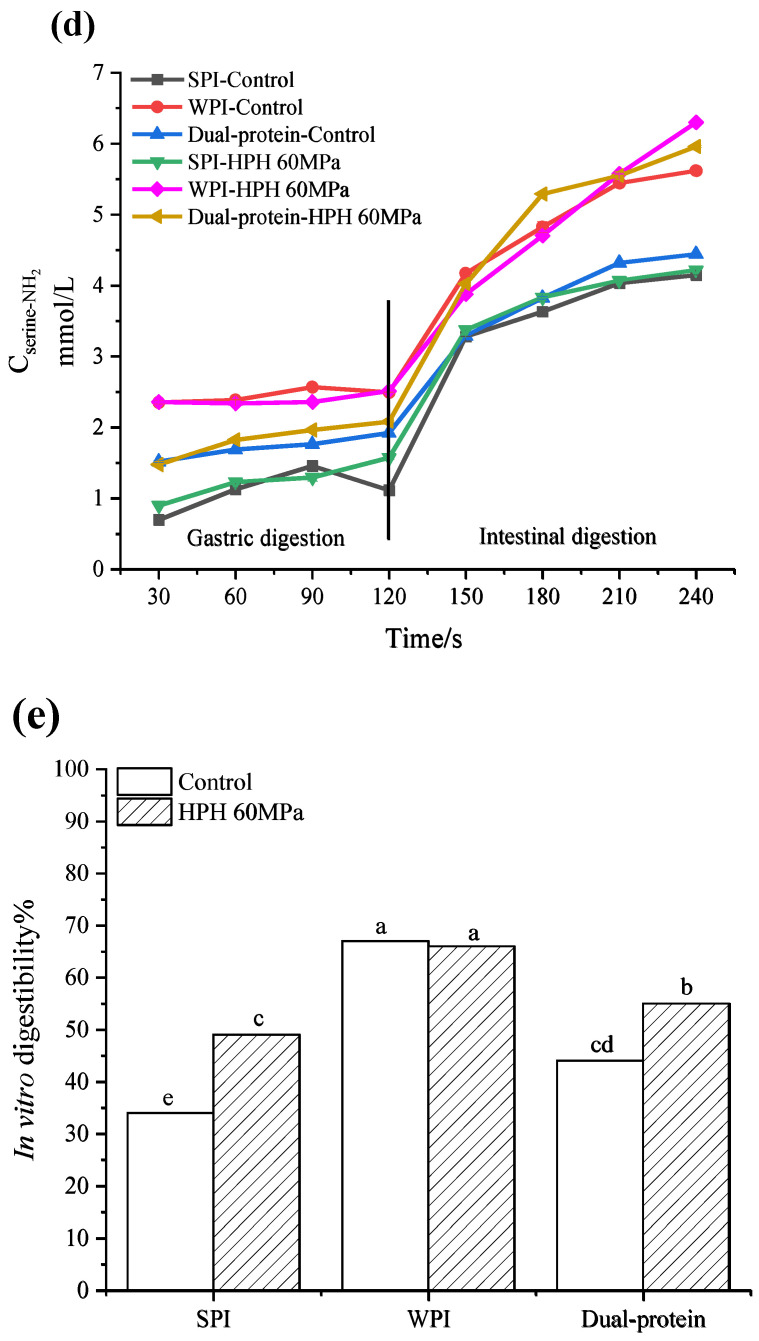
The particle size and zeta potential of SPI (**a**), WPI (**b**) and dual-protein (**c**) emulsions containing a 10% (*w*/*w*) oil phase during in vitro digestion, as well as the free amino groups (-NH_2_) (**d**) and in vitro digestibility (**e**) of protein emulsions containing a 10% (*w*/*w*) oil phase. Different lowercase letters in Figures (**a**–**c**,**e**) indicate significant differences in the different protein emulsions.

Changes in the droplet size and zeta potential during the in vitro digestion of the SPI, WPI and dual-protein emulsions containing a 10% (*w*/*w*) oil phase concentration are shown in Figure 5a–c, respectively. It can be observed that the zeta potentials of all the protein emulsions decreased significantly during gastric digestion, which was due to the high concentration of salt ions in the gastric digestive fluid and because the low-pH environment reduced the strength of the electrostatic repulsion between droplets and accelerated the droplets’ aggregation [38]. The droplet size of all the SPI and dual-protein emulsions obviously increased in gastric digestion, and the droplet size of the HPH-modified dual-protein emulsion was larger than the untreated one, which might be attributed to the complex interface protein composition that decreased the stability in the gastric digestion phase. Contrarily, the droplet size of the WPI emulsion did not increase significantly during gastric digestion; perhaps the β-lactoglobulin (β-Lg) in the WPI had a certain resistance to pepsin that was not easy to hydrolyze, so the droplet size did not obviously change [39].

In the intestinal digestion phase, the zeta potentials of all the protein emulsions were significantly higher than in the gastric digestion phase because the pH environment during intestinal digestion (7.0) was much higher than the isoelectric point of the protein, which was beneficial for increasing the net charge on the surfaces of the droplets to improve the electrostatic balance between the droplets [40]. The droplet size of all the SPI and dual-protein emulsions obviously decreased in the initial period of the intestinal digestion phase, while the droplet size of the dual-protein emulsion increased again after 60 min of intestinal digestion, and the same phenomenon could be found in the WPI emulsion; perhaps it was related to the aggregation of large particles, such as free fatty acids and other digestive products [41]. Moreover, the droplet size of the HPH-modified dual-protein emulsion in the terminal period of intestinal digestion was lower than the untreated one, indicating that the degree of digestion and the decomposition of the dual-protein emulsion improved after HPH treatment.

#### 3.5.2. The Release of Free Amino Groups during In Vitro Digestion

The release of free amino groups (-NH_2_) could reflect the degree of protein digestion in the gastrointestinal tract. The higher the concentration of released -NH_2_, the greater the degree of protein digestion and hydrolysis [42]. Figure 5d shows the release of free amino groups from the protein emulsions during in vitro digestion. In gastric digestion, the rising trends of the release of free amino groups of all the protein emulsions were slow; however, the release of free amino groups rose significantly at 30 min of intestinal digestion and kept rising in subsequent intestinal digestion. The release of free amino groups in the HPH-modified SPI emulsion had no significant difference compared to the untreated one, but the release of free amino groups of the WPI and dual-protein emulsions increased further after HPH treatment. In particular, the increasing trend of the HPH-modified dual-protein emulsion was significantly higher than that of the HPH-modified WPI emulsion compared to the control, indicating that HPH had a better effect on promoting the digestion and hydrolysis of the dual-protein emulsion. 

#### 3.5.3. In Vitro Digestibility

The in vitro digestibility of the emulsions is shown in Figure 5e. It can be seen that after HPH treatment, the in vitro digestibility of the SPI and dual-protein emulsions significantly increased from 34% to 49% and from 44% to 55%, respectively. These results might be attributed to the disruptions in non-covalent and covalent bonds that caused the structure of protein to unfold after HPH treatment, making it easier for digestive enzymes to attack and combine with enzyme digestion sites. However, the in vitro digestibility of the HPH-modified WPI emulsion did not increase, which could be related to disulfide-bond-mediated protein aggregation that reduced the accessibility of digestive enzymes [43].

### 3.6. Storage Stability

**Figure 6:**
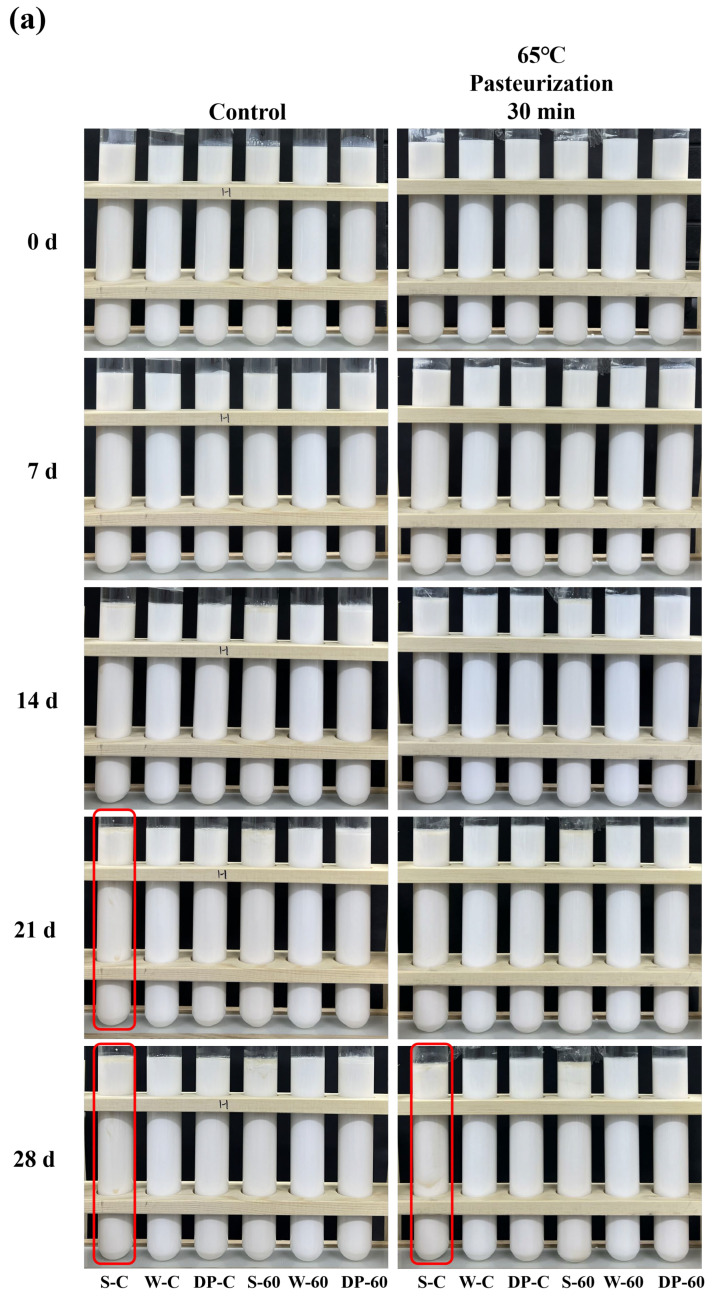

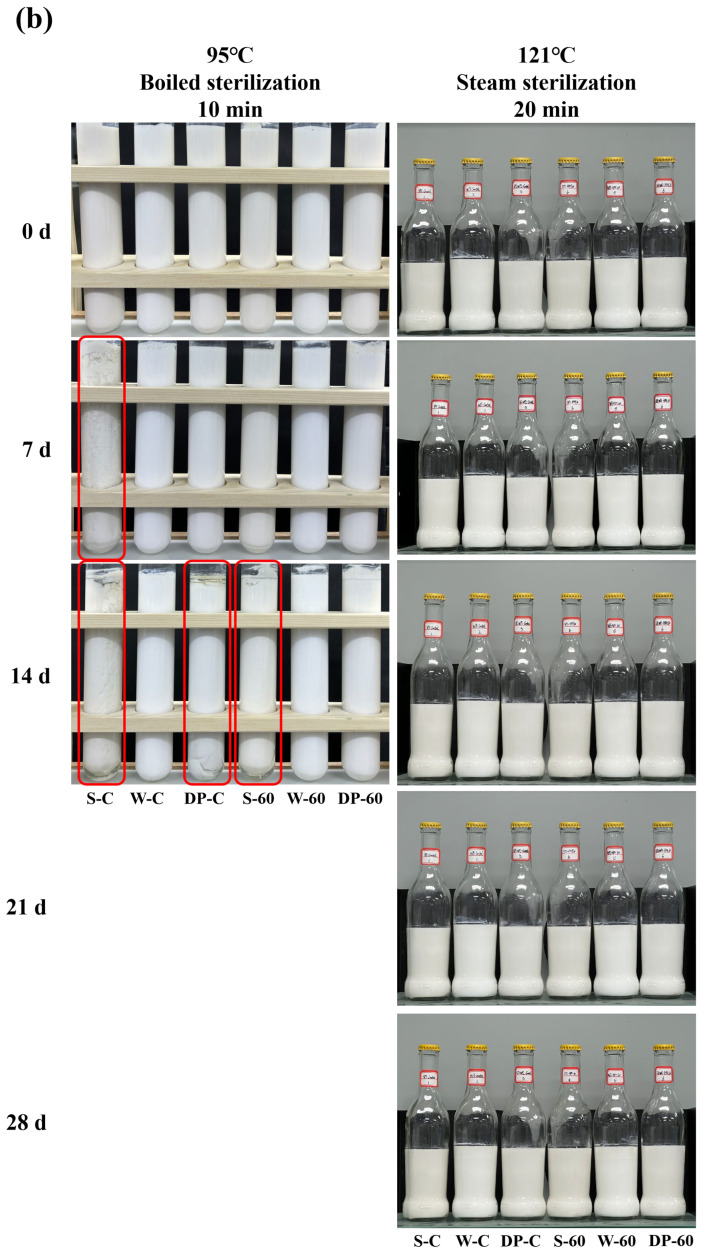
The appearances of protein emulsions containing different oil phase concentrations under different sterilization methods during low-temperature (4 °C) storage (**a**) and room-temperature (25 °C) storage (**b**). The “S-C”, “W-C”, “DP-C”, “S-60”, “W-60”, and “DP-60” in Figure (**a**,**b**) indicate “SPI-control”, “WPI-control”, “Dual-protein-control”, “SPI-HPH 60 MPa”, “WPI-HPH 60 MPa”, and “Dual-protein-HPH 60 MPa”, respectively.

Changes in the appearances of the protein emulsions under different sterilization and storage temperatures are shown in Figure 6a,b. In low-temperature storage (4 °C), it can be seen that there was no significant change in any of the samples within 14 d. As storage time increased, a slight oil–water stratification appeared on the untreated SPI emulsion in the control group at both 21 and 28 d. However, in the pasteurization group, the stratification phenomenon in the untreated SPI emulsion only happened at 28 d, therefore indicating that pasteurization combined with low-temperature storage could significantly improve the storage stability of an untreated SPI emulsion. Beyond that, the other protein samples maintained a uniform state during 28 d of storage.

In room-temperature (25 °C) storage, obvious flocculation appeared in the untreated SPI emulsion at 7 d in the boiled-sterilization group, and this phenomenon also appeared in the untreated dual-protein emulsion and the HPH-modified SPI emulsion in the same group at 14 d; it might be attributed to the microorganisms remaining in the emulsion growing and reproducing, which caused the emulsion flocculation. It was worth noting no flocculation happened in the HPH-modified dual-protein emulsion after boiled sterilization within 14 d, suggesting that HPH could improve the antibacterial effect in a dual-protein emulsion to a certain extent. Since the microorganism contamination in the boiled-sterilization emulsions gradually increased after 14 d at room-temperature storage, which we could no longer carry on observing, the appearance changes of the emulsions are shown for only 14 d. Oppositely, the appearances of the emulsions after steam sterilization did not change significantly within 28 d of room-temperature storage. By comparison, it was found that the HPH-modified dual-protein emulsion had the best stability after steam sterilization during the period of room-temperature storage.

## 4. Conclusions

This study researched the emulsifying properties, in vitro digestion characteristics, and storage stability of HPH-modified dual-protein emulsions. The research results show that increasing the oil phase concentration increased the droplet size and the absorbed protein at the O/W interface of the dual-protein emulsion; meanwhile, it also improved the viscoelasticity of the dual-protein emulsion. It could be found that when the oil phase concentration was 10% (*w*/*w*), the diffusion stability and AP% of the HPH-modified dual-protein emulsion were both significantly better than the control, and the emulsion elasticity was enhanced, showing a more significant viscous liquid behavior than a single-protein emulsion. The in vitro digestion results indicate that HPH treatment obviously improved the in vitro digestibility of a dual-protein emulsion containing a 10% (*w*/*w*) oil phase concentration, promoting the release of free amino groups during in vitro digestion, and the degree of digestion and the decomposition of the emulsion in the gastrointestinal tract significantly improved. In addition, the storage period of the dual-protein emulsion was effectively extended after HPH treatment, demonstrating better storage stability than a single-protein emulsion after steam sterilization and room-temperature storage. This study has great significance for the in-depth development and utilization of HPH in liquid dual-protein products, and it also enriches the research content on the application of non-thermal processing, especially HPH technology, in animal–plant mixed proteins, which is helpful for using them in the food industry and much more.

## Figures and Tables

**Table 1:** The TSI values of protein emulsions containing different oil phase concentrations.

Oil Phase Concentration (%, *w*/*w*)	Control			HPH 60 MPa		
	SPI	WPI	Dual Protein	SPI	WPI	Dual Protein
5	0.2	0.9	0.7	0.3	1.2	0.8
10	0.2	0.4	0.2	0.1	0.6	0.3
15	0.5	0.6	0.4	0.8	0.7	0.5

## Data Availability

The data presented in this study are available upon request from the corresponding author.

## References

[1] McClements D.J., Rao J.J. (2011). Food-grade nanoemulsions: Formulation, fabrication, properties, performance, biological fate, and potential toxicity. Crit. Rev. Food Sci. Nutr..

[2] Sivapratha S., Sarkar P. (2018). Multiple layers and conjugate materials for food emulsion stabilization. Crit. Rev. Food Sci. Nutr..

[3] Dickinson E. (2008). Interfacial structure and stability of food emulsions as affected by protein-polysaccharide interactions. Soft Matter.

[4] Rutkevicius M., Allred S., Velev O.D., Velikov K.P. (2018). Stabilization of oil continuous emulsions with colloidal particles from water-insoluble plant proteins. Food Hydrocoll..

[5] Yan X.J., Ma C.C., Cui F.Z., McClements D.J., Liu X.B., Liu F.G. (2020). Protein-stabilized pickering emulsions: Formation, stability, properties, and applications in foods. Trends Food Sci. Technol..

[6] Bos M.A., Van Vliet T. (2001). Interfacial rheological properties of adsorbed protein layers and surfactants: A review. Adv. Colloid Interface Sci..

[7] Henchion M., Hayes M., Mullen A.M., Fenelon M., Tiwari B. (2017). Future protein supply and demand: Strategies and factors influencing a sustainable equilibrium. Foods.

[8] Alves A.C., Tavares G.M. (2019). Mixing animal and plant proteins: Is this a way to improve protein techno-functionalities?. Food Hydrocoll..

[9] Ren G.X., Zhang J.P., Li M.H., Yi S.Q., Wang J. (2017). Protein blend ingestion before allogeneic stem cell transplantation improves protein-energy malnutrition in patients with leukemia. Nutr. Res..

[10] Zhang J.J., Zhang Q.W., Liu H., Liu X.Y., Yu Y.H., Han D., He X.Y., Zeng P., Wang J. (2022). Soy-whey dual-protein alleviates osteoporosis of ovariectomized rats via regulating bone fat metabolism through gut-liver-bone axis. Nutrition.

[11] Huang Y.C., Zhang K., Zhang L., Qiu J.H., Fu L., Ying T.Y., Wang J., Qin R., Zhang J.J., Dong X.W. (2022). Dosage of dual-protein nutrition differentially impacts the formation of atherosclerosis in apoE-/-mice. Nutrients.

[12] Nishinari K., Fang Y., Guo S., Phillips G.O. (2014). Soy proteins: A review on composition, aggregation and emulsification. Food Hydrocoll..

[13] Wagner J., Biliaderis C.G., Moschakis T. (2020). Whey proteins: Musings on denaturation, aggregate formation and gelation. Crit. Rev. Food Sci. Nutr..

[14] Tang C.H. (2019). Nanostructured soy proteins: Fabrication and applications as delivery systems for bioactives (a review). Food Hydrocoll..

[15] Szczepanska J., Skapska S., Polaska M., Marszalek K. (2022). High pressure homogenization with a cooling circulating system: The effect on physiochemical and rheological properties, enzymes, and carotenoid profile of carrot juice. Food Chem..

[16] Kruszewski B., Domian E., Nowacka M. (2023). Influence of high-pressure homogenization on the physicochemical properties and betalain pigments of red beetroot (*Beta vulgaris* L.) juice. Molecules.

[17] Dumay E., Chevalier-Lucia D., Picart-Palmade L., Benzaria A., Gracia-Julia A., Blayo C. (2013). Technological aspects and potential applications of (ultra) high-pressure homogenisation. Trends Food Sci. Technol..

[18] Sahil, Madhunita M., Prabhakar P.K., Kumar N. (2022). Dynamic high pressure treatments: Current advances on mechanistic-cumtransport phenomena approaches and plant protein functionalization. Crit. Rev. Food Sci. Nutr..

[19] Ma W.C., Wang J.M., Wu D., Xu X.B., Wu C., Du M. (2019). Physicochemical properties and oil/water interfacial adsorption behavior of cod proteins as affected by high-pressure homogenization. Food Hydrocoll..

[20] Fernandez-Avila C., Trujillo A.J. (2016). Ultra-high pressure homogenization improves oxidative stability and interfacial properties of soy protein isolate-stabilized emulsions. Food Chem..

[21] Melchior S., Moretton M., Calligaris S., Manzocco L., Nicoli M.C. (2022). High pressure homogenization shapes the techno-functionalities and digestibility of pea proteins. Food Bioprod. Process..

[22] Dybowska B.E., Krupa-Kozak U. (2020). Stability of oil-in-water emulsions as influenced by thermal treatment of whey protein dispersions or emulsions. Int. J. Dairy Technol..

[23] Lunardi C.N., Gomes A.J., Rocha F.S., Tommaso J.D., Patience G.S. (2020). Experimental methods in chemical engineering: Zeta potential. Can. J. Chem. Eng..

[24] Sun S., Li S.H., Yan H.J., Zou H.N., Yu C.P. (2022). The conformation and physico-chemical properties of pH-treated golden pompano protein on the oil/water interfacial properties and emulsion stability. Int. J. Food Sci. Technol..

[25] Shi J.Y., Xiao J.X., Liu L., Dong X.Y. (2021). Ultrasonic assisted oil-in-water emulsions stabilized by flaxseed protein isolate: Influence of different oils. J. Dispers. Sci. Technol..

[26] Zhu Q., Qiu S., Zhang H., Cheng Y., Yin L. (2018). Physical stability, microstructure and micro-rheological properties of water-in-oil-in-water (W/O/W) emulsions stabilized by porcine gelatin. Food Chem..

[27] Ding M.Z., Liu L.J., Zhang T., Tao N.P., Wang X.C., Zhong J. (2021). Effect of interfacial layer number on the storage stability and in vitro digestion of fish oil-loaded multilayer emulsions consisting of gelatin particle and polysaccharides. Food Chem..

[28] Church F.C., Swaisgood H.E., Porter D.H., Catignani G.L. (1983). Spectrophotometric assay using o-phthaldialdehyde for determination of proteolysis in milk and isolated milk proteins. J. Dairy Sci..

[29] Gupta A., Badruddoza A.Z.M., Doyle P.S. (2017). A general route for nanoemulsion synthesis using low-energy methods at constant temperature. Langmuir ACS J. Surf. Colloids.

[30] Song X.Z., Zhou C.J., Fu F., Chen Z.L., Wu Q.L. (2013). Effect of high-pressure homogenization on particle size and film properties of soy protein isolate. Ind. Crops Prod..

[31] Liu F., Tang C.H. (2016). Soy glycinin as food-grade pickering stabilizers: Part. I. structural characteristics, emulsifying properties and adsorption/arrangement at interface. Food Hydrocoll..

[32] Zhang Y.H., Zhou F.B., Shen P.H., Zhao Q.Z., Zhao M.M. (2020). Influence of thermal treatment on oil-water interfacial properties and emulsion stabilization prepared by sono-assembled soy peptide nanoparticles. Food Hydrocoll..

[33] Christian C., Elena T., Donato C., Donatella P., Massimo F. (2019). Turbiscan Lab^®^ expert analysis of the stability of ethosomes^®^ and ultradeformable liposomes containing a bilayer fluidizing agent. Colloids Surf. B Biointerfaces.

[34] Hebishy E., Ferragut V., Blasco-Moreno A., Trujillo A.J. (2019). Impact of oil phase concentration on physical and oxidative stability of oil-in-water emulsions stabilized by sodium caseinate and ultra-high pressure homogenization. J. Dispers. Sci. Technol..

[35] Yang J.Q., Liu G.Y., Zeng H.B., Chen L.Y. (2018). Effects of high pressure homogenization on faba bean protein aggregation in relation to solubility and interfacial properties. Food Hydrocoll..

[36] Meleties M., Martineau R.L., Gupta M.K., Montclare J.K. (2022). Particle-based microrheology as a tool for characterizing protein based materials. ACS Biomater. Sci. Eng..

[37] Tisserand C., Fleury M., Brunel L., Bru P., Meunier G. (2012). Analytical series soft matter analysis by means of microrheology. JCT CoatingsTech.

[38] Li Q., He S.H., Xu W.L., Peng F.S., Gu C., Wang R.C., Ma Y. (2018). Formation, stability and in vitro digestion of beta-carotene in oil-in-water milk fat globule membrane protein emulsions. Food Biophys..

[39] Ozel B., Zhang Z.Y., He L.L., McClements D.J. (2020). Digestion of animal-and plant-based proteins encapsulated in κ-carrageenan/protein beads under simulated gastrointestinal conditions. Food Res. Int..

[40] Zhang R.J., Zhang Z.P., Zhang H., Decker E.A., McClements D.J. (2015). Influence of lipid type on gastrointestinal fate of oil-in-water emulsions: In vitro digestion study. Food Res. Int..

[41] Yao M.F., Xiao H., McClements D.J. (2014). Delivery of lipophilic bioactives: Assembly, disassembly, and reassembly of lipid nanoparticles. Annu. Rev. Food Sci. Technol..

[42] Nielsen P.M., Petersen D., Dambmann C. (2001). Improved method for determining food protein degree of hydrolysis. J. Food Sci..

[43] Sun M.J., Mu T.H., Sun H.N., Zhang M. (2014). Digestibility and structural properties of thermal and high hydrostatic pressure treated sweet potato (*Ipomoea batatas* L.) protein. Plants Foods Hum. Nutr..

